# Osteoporosis screening using X-ray assessment and osteoporosis self-assessment tool for Asians in hip surgery patients

**DOI:** 10.1007/s00774-024-01569-5

**Published:** 2024-12-10

**Authors:** Ryo Higuchi, Keisuke Uemura, Sotaro Kono, Hirokazu Mae, Kazuma Takashima, Hirohito Abe, Takashi Imagama, Takashi Sakai, Seiji Okada, Hidetoshi Hamada

**Affiliations:** 1 Department of Orthopaedics, Osaka University Graduate School of Medicine, 2-2 Yamadaoka, Suita, Osaka 565-0871 Japan; 2 Department of Orthopaedic Medical Engineering, Osaka University Graduate School of Medicine, 2-2 Yamadaoka, Suita, Osaka 565-0871 Japan; 3 Department of Orthopaedics, Japan Community Health Care Organization Hoshigaoka Medical Center, 4-8-1, Hoshigaoka, Hirakata, Osaka 573-0013 Japan; 4 Department of Orthopaedics, Yamaguchi University Graduate School of Medicine, 1-1-1, Minamikogushi, Ube, Yamaguchi 755-0046 Japan

**Keywords:** Bone mineral density, Hip arthroplasty, Osteoporosis, Proximal femoral fracture, Screening

## Abstract

**Objectives:**

As many patients with osteoporosis remain undiagnosed, we aimed to develop a simple method to efficiently screen for osteoporosis using a combination of anteroposterior hip X-ray assessment and the Osteoporosis Self-Assessment Tool for Asians (OSTA), which is calculated as (body weight − age) × 0.2.

**Methods:**

One hundred Japanese women (age: 73 ± 11 years, body weight: 54.4 ± 11.1 kg) who underwent hip surgery, anteroposterior hip X-ray, and DXA were included. Based on the DXA results of the total proximal femur, 35 cases were diagnosed with osteoporosis. Fifteen orthopaedic surgeons visually inspected the hip X-ray images and scored the suspicion of osteoporosis on a scale of 1–4 (1: very unlikely, 4: very suspicious), which is referred to as “pred-score.” In addition, OSTA was calculated as a continuous variable (OSTA score). Osteoporosis was screened using the pred-score and OSTA score, and both scores were analyzed using the receiver operating characteristic curves.

**Results:**

The area under the curves (AUCs) of the pred-score and OSTA score were 0.626–0.875 and 0.817 across surgeons, respectively. When both scores were used, the AUC for screening osteoporosis ranged from 0.821 to 0.915 across surgeons. Significant improvement from AUCs calculated with the pred-score or OSTA score was found in 11 surgeons (73.3%).

**Conclusion:**

The combination of X-ray assessment and OSTA can be used to screen for osteoporosis and has the potential to be used as a new simple screening tool in daily clinical practice.

## Introduction

The bone mineral density (BMD) of the proximal femur plays an important role in the selection of hip implants (e.g., cement or cementless stem) and initial fixation of implants for hip arthroplasty [1, 2]. A low BMD is reported to be a risk factor for intraoperative periprosthetic fractures [3]. Thus, the maintenance of BMD after surgery is important to prevent stem subsidence and periprosthetic fractures, which drastically decrease the activity level and quality of life of patients [1]. Moreover, adequate diagnosis and subsequent treatment for osteoporosis are essential in patients undergoing hip surgery to maintain good clinical outcomes.

Osteoporosis is usually diagnosed based on the measurement of BMD of the lumbar spine and/or proximal femur using dual-energy X-ray absorptiometry (DXA), which is considered as the gold standard [4, 5]. However, because of its cost and limited availability, preoperative DXA assessment cannot always be performed for patients undergoing hip surgery or during their annual follow-up, particularly in large hospitals where DXA examination time slots are limited compared with the number of outpatient patients. Hence, patients with a high risk of osteoporosis need to be effectively selected for further examination using DXA.

The findings of hip X-rays can be used to aid in efficiently selecting patients with a high risk of osteoporosis. Surgeons have widely used parameters, including the Singh index [6], cortical thickness index [7], canal flare index [8], canal-to-calcar ratio [7], and Dorr classification [7], to estimate the BMD of the proximal femur. In fact, some studies have reported the potential use of these parameters for osteoporosis screening [9–12].

Other tools that may be used for osteoporosis screening include self-screening tools, such as the Simple Calculated Osteoporosis Risk Estimation (SCORE) [13]; Osteoporosis Risk Assessment Instrument (ORAI) [14]; Age, Bulk, One or Never Estrogens (ABONE) [15]; and Osteoporosis Self-Assessment Tool for Asians (OSTA) [16]. Among them, OSTA is the simplest tool. Originally developed for Asian postmenopausal women, this tool only uses two variables and is calculated using the following equation: (body weight − age) × 0.2. The cut-off value of −1 indicates a threshold for low risk of osteoporosis, whereas −4 indicates a high risk. While there is a need to modify the cut-off value, the usefulness of OSTA has been verified in several Asian countries [16–22], including Japan [23].

Studies have shown the efficacy of each tool in osteoporosis screening. Thus, we hypothesized that osteoporosis could be more efficiently screened using a combination of X-ray findings and self-screening tools, allowing DXA examinations to be performed on patients more likely to have osteoporosis. Therefore, the present study aimed to (1) develop and verify a simple screening method by combining surgeon’s hip X-ray assessment and OSTA and to (2) analyze whether surgeons’ experience affects its predictive value in order to confirm the generalizability and facilitate the use of this combined method in daily clinical practice.

## Materials and methods

### Participants

Ethical approval was obtained from the institutional review boards of all institutions participating in this retrospective study. Informed consent was obtained from all patients in the form of opt-out. One hundred women aged over 40 years who underwent hip surgery at two institutions (A and B, with 50 cases each) were included in this study (Table 1). The age, height, weight, and body mass index of participants (presented as mean ± standard deviation [SD]) were as follows: 73.2 ± 10.2 years, 152.0 ± 5.6 cm, 54.1 ± 10.6 kg, and 23.3 ± 4.1 kg/m^2^, respectively. Eighty patients underwent elective surgery (i.e., total hip arthroplasty), whereas 20 patients underwent surgery for proximal femoral fracture (Table 1). Among them, the non-operated side with no obvious osteoarthritis or deformity was used for analysis.

**Table 1:** Demographic and clinical characteristics of patients from institutions A and B

Factors	A	B	Overall
Number of cases	50	50	100
Disease (operated side)	OA: 45 RDC: 3 ONFH: 2	OA: 28 PFF: 20 ONFH: 1 Stem revision: 1	OA: 73 PFF: 20 RDC: 3 ONFH: 3 Stem revision: 1
Age (years)	68.2 ± 9.5	78.2 ± 8.4	73.2 ±10.2
Height (cm)	152.6 ± 5.3	151.4 ± 5.9	152.0 ± 5.6
Weight (kg)	55.8 ± 9.8	52.3 ± 11.1	54.1 ±10.6
BMI (kg/m^2^)	24.0 ± 4.2	22.7 ± 4.0	23.3 ± 4.1
DXA-BMD (g/cm^2^)	0.722 ± 0.137	0.657 ± 0.167	0.689 ± 0.155
Osteoporosis	11	24	35 (35%)

*BMI* body mass index, *OA* osteoarthritis, *RDC* rapidly destructive coxopathy, *ONFH* osteonecrosis of the femoral head, *PFF* proximal femoral fracture, *DXA* dual-energy X-ray absorptiometry, *BMD* bone mineral density

### DXA acquisition and diagnosis of osteoporosis

All patients underwent BMD measurement of the proximal femur of the contralateral side (i.e., nonsurgical side) before or after surgery using DXA to select the appropriate surgical implant and initiate osteoporosis treatment when diagnosed. Hologic’s DXA was used in both institutions (A: Horizon A, B: Horizon Wi). DXA calibration was performed at each institution each day using a phantom provided by the manufacturer. Osteoporosis was diagnosed on the basis of a T-score of −2.5 at the proximal femoral region (i.e., total region), which was calculated on the basis of the guidelines of the International Society for Clinical Densitometry [24] with modifications for Japanese subjects [4].

### Relationship between surgeons’ X-ray assessment and BMD of the total proximal femur region measured by DXA (DXA-BMD) and osteoporosis

**Fig. 1:**
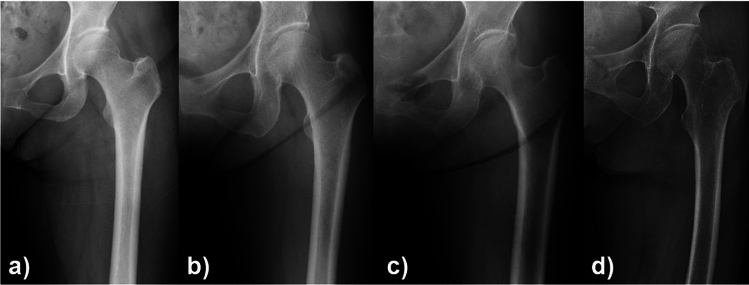
Examples of four X-rays used to classify osteoporosis likelihood. The ave.pred-score of 15 surgeons for these cases were **a** 1.1, **b** 2.0, **c** 2.9, and **d** 3.9

Fifteen orthopedic surgeons, including five board-certified surgeons (staff surgeons) and 10 residents (less than four years of experience in orthopaedics), participated in this study. First, a surgeon who did not participate in the X-ray assessment selected the anteroposterior hip X-ray and created the material for assessment. The surgeon was blinded with regard to the operated side and patient demographics (e.g., age and hip disease) in order to avoid bias (Fig. 1a–d). Then, each surgeon visually inspected the hip X-rays and scored the suspicion of osteoporosis on a scale of 1–4 (1: very unlikely (Fig. 1a), 2: unlikely (Fig. 1b), 3: suspicious (Fig. 1c), and 4: very suspicious (Fig. 1d)) for each case (pred-score). The average pred-score of all surgeons for each case was also calculated (ave.pred-score). Interobserver and intraobserver agreement was assessed because visual inspection is subjective. Specifically, eight surgeons (four staff surgeons and four residents) visually reassessed the hip X-rays of 100 patients with an interval of more than two weeks.

The relationship between the ave.pred-score and DXA-BMD was analyzed. Moreover, the relationships between pred-score and osteoporosis were analyzed for each surgeon and for all surgeons (15 surgeons). Furthermore, the relationships were calculated for staff surgeons and residents and compared to assess whether the surgeon’s experience affected the predictive accuracy.

### Relationship between OSTA score and DXA-BMD and osteoporosis

OSTA was calculated as follows: (body weight − age) × 0.2. It was evaluated as a continuous variable and referred to as the “OSTA score.” The relationship between OSTA score and DXA-BMD and osteoporosis was analyzed.

### Data analyses

The Pearson correlation coefficient was used to assess the correlation between DXA-BMD and surgeons’ X-ray assessment (ave.pred-score) and OSTA score. The relationship between the pred-score and OSTA score in diagnosing osteoporosis was assessed using receiver operating characteristic (ROC) curve analysis. A combined ROC curve analysis was performed using the pred-score and OSTA score to predict osteoporosis. The area under the curve (AUC), specificity, sensitivity, and cut-off value were quantified for each ROC curve analysis. AUCs of 0.61–0.80, 0.81–0.90, and >0.90 were considered moderate, good, and excellent, respectively [25]. Interobserver and intraobserver agreement in X-ray assessment was evaluated using the quadratic weighted kappa coefficient. Kappa values of 0.81–1.00 were considered almost perfect [26]. All statistical analyses were performed using JMP Pro 17 (SAS Institute Japan, Tokyo, Japan) and MATLAB v9.10 (MathWorks, Natick, MA, USA). Statistical significance was considered at *p* < 0.05.

## Results

### Results of ave.pred-score, DXA-BMD, and OSTA score

The mean (± SD) ave.pred-score, DXA-BMD, and OSTA score were 2.3 ± 0.8, 0.689 ± 0.155 g/cm^2^, and −3.8 ± 3.4, respectively. Osteoporosis was diagnosed in 35 patients based on a T-score < −2.5 (Table 1).

### Relationship between surgeons’ X-ray assessment and DXA-BMD and osteoporosis

**Fig. 2:**
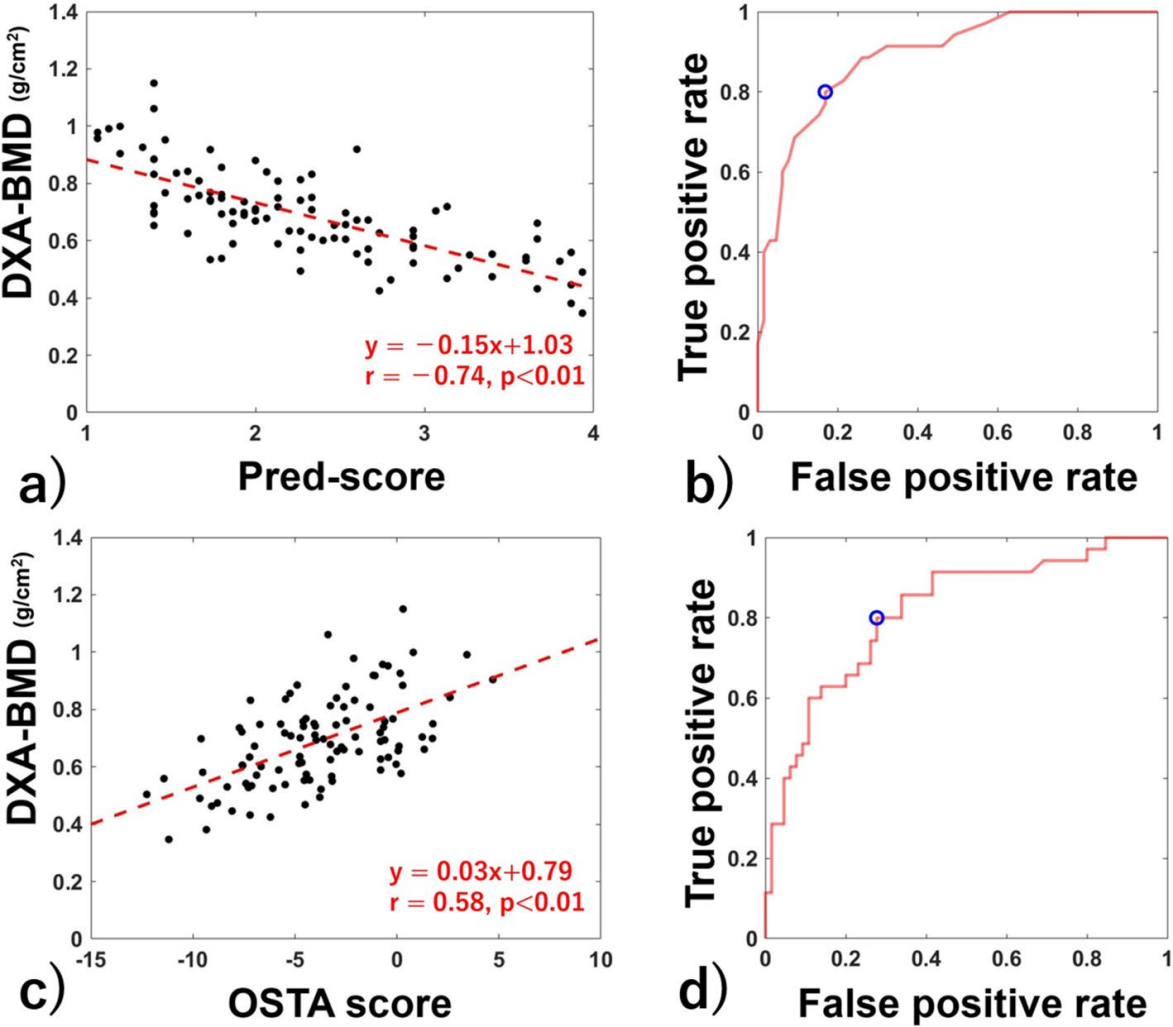
Correlation of each score between DXA-BMD and the ROC curve for diagnosing osteoporosis by each score. Correlation between pred-score and DXA-BMD (**a**) and ROC curve for diagnosing osteoporosis using the pred-score (**b**). Correlation between OSTA score and DXA-BMD (**c**) and ROC curve for diagnosing osteoporosis using the OSTA score (**d**). The red dotted line in (**a**) and (**c**) indicates the regression line, and red letters indicate the regression equation, coefficient of determination, and *p*-value. The blue circle in (**b**) and (**d**) indicates the optimal cut-off point

The correlation between the ave.pred-score and DXA-BMD was *r* = −0.74 (*p* < 0.01) (Fig. 2a). In the ROC curve analysis to predict osteoporosis based on the ave.pred-score, an optimal ave.pred-score cut-off value of 2.4 showed 80.0% sensitivity and 83.1% specificity (AUC: 0.890) (Fig. 2b). When compared across surgeons, the AUCs ranged from 0.626 to 0.875, which were significantly different across surgeons (*p* < 0.01) (Table 2). The AUC using the staff surgeons’ pred-score was 0.908, which was not statistically different from that of residents (0.871, *p* = 0.07) (Table 2).

**Table 2:** Results of ROC curve analysis to predict osteoporosis using the pred-score across surgeons and surgeon groups

	Cut-off value	AUC	Sensitivity (%)	Specificity (%)
Staff surgeons				
A	2	0.764	60.0	92.3
B	3	0.873	68.6	92.3
C	2	0.875	97.1	60.0
D	3	0.844	68.6	84.6
E	3	0.820	74.3	77.0
Residents				
F	3	0.868	85.7	76.9
G	3	0.626	51.4	72.3
H	3	0.784	85.7	62.5
I	3	0.774	65.7	80.0
J	3	0.811	71.4	73.8
K	3	0.666	77.1	52.3
L	4	0.758	45.7	95.4
M	3	0.781	62.9	78.5
N	3	0.821	82.9	66.2
O	3	0.793	74.3	69.2
Staff surgeons overall	2.2	0.908	80.0	89.2
Residents overall	2.5	0.871	85.7	75.4
Overall	2.4	0.890	80.0	83.1

*AUC* area under the curve

### Relationship between OSTA score and DXA-BMD and osteoporosis

The correlation between the OSTA score and DXA-BMD was *r* = 0.58 (*p* < 0.01) (Fig. 2c). In the ROC curve analysis to predict osteoporosis based on the OSTA score, an optimal OSTA score cut-off value of −4.3 showed 80.0% sensitivity and 72.3% specificity (AUC: 0.817) (Fig. 2d).

### Combined analysis of the relationship between surgeons’ X-ray assessment and OSTA score and osteoporosis

**Fig. 3:**
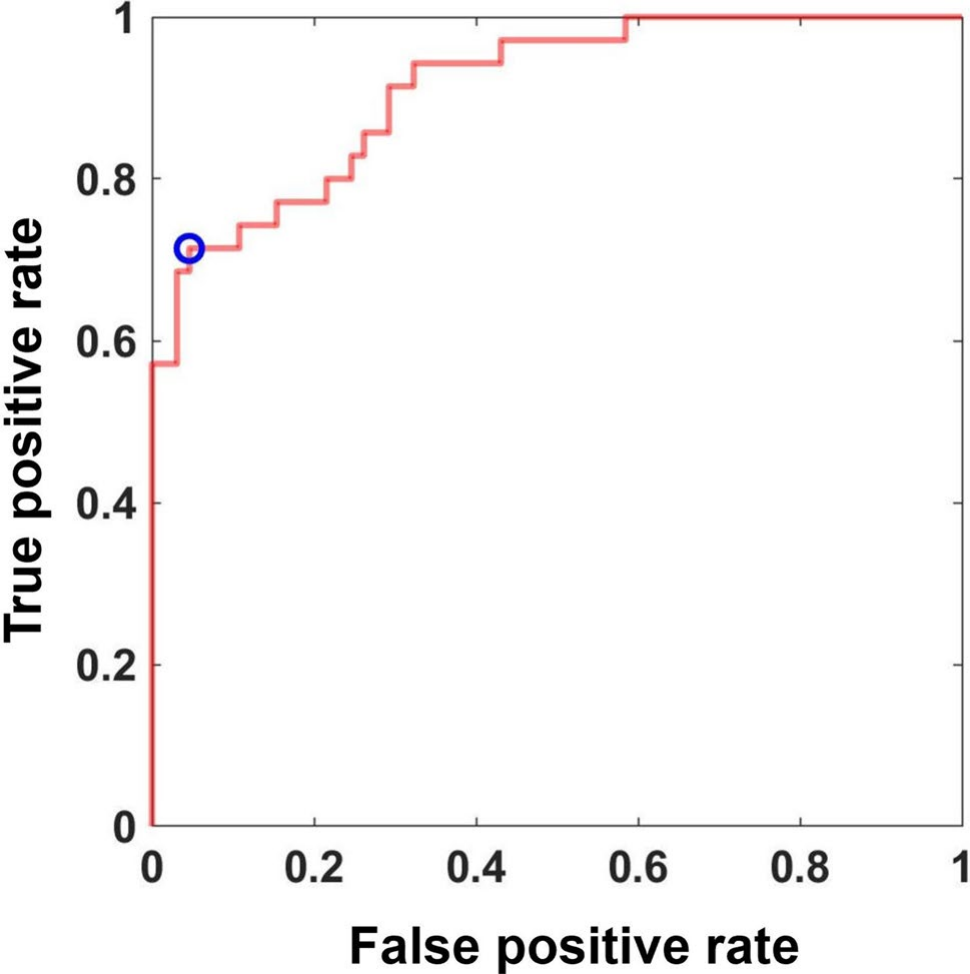
ROC curve for diagnosing osteoporosis using the pred-score and OSTA score. The blue circle indicates the optimal cut-off point

In the ROC curve analysis to predict osteoporosis based on the ave.pred-score and OSTA score, the AUC was 0.912 with a sensitivity of 71.4% and specificity of 95.4% (Fig. 3). When compared across surgeons, the AUCs ranged from 0.821 to 0.915, which were significantly different across surgeons (*p* < 0.01) (Table 3). The sensitivity ranged between 66.2 and 85.7%, whereas the specificity ranged between 74.6 and 92.3% (Table 3). The AUCs significantly improved from that calculated using only the pred-score for eight surgeons (53.3%) or that using only the OSTA score for seven surgeons (46.7%), resulting in an increase of 73.3% in 11 surgeons when only one parameter (pred-score or OSTA score) was used (Table 3). The AUC using the staff surgeons’ pred-score was 0.925, which was not statistically different from that of residents (0.896, *p* = 0.09).

**Table 3:** Results of ROC curve analysis to predict osteoporosis using the pred-score and OSTA score across surgeons and surgeon groups

	AUC	Sensitivity (%)	Specificity (%)
Staff surgeons			
A	0.879*	80.0	74.6
B	0.906^#^	85.7	81.5
C	0.909^#^	80.0	86.2
D	0.903*^#^	71.4	92.3
E	0.878^#^	82.9	75.4
Residents			
F	0.915*^#^	77.1	90.7
G	0.821*	74.3	80.0
H	0.847	66.2	91.4
I	0.833	71.4	83.1
J	0.865	74.3	87.7
K	0.832*	77.1	80.0
L	0.857*	74.3	84.6
M	0.894*^#^	74.3	84.6
N	0.882*^#^	80.0	74.6
O	0.850	77.1	80.0
Staff surgeons overall	0.925^#^	88.6	84.6
Residents overall	0.896^#^	71.4	93.8
Overall	0.912^#^	71.4	95.4

*OSTA* Osteoporosis Self-Assessment Tool for Asians, *AUC* area under the curve

^*^Statistically higher than the AUC calculated using the pred-score

^#^Statistically higher than the AUC calculated using the OSTA score

### Interobserver and intraobserver agreement in surgeons’ X-ray assessment

The kappa coefficient of the pred-score across surgeons (i.e., interobserver agreement) ranged between 0.868 and 0.967, whereas that within surgeons (i.e., intraobserver agreement) ranged between 0.762 and 0.963 (Table 4).

**Table 4:** Intraobserver agreement of the pred-score for eight surgeons

	Staff surgeons				Residents			
	A	B	C	D	F	G	I	J
Kappa coefficient	0.901	0.967	0.959	0.949	0.930	0.923	0.938	0.868

## Discussion

We used a combination of surgeon’s X-ray assessment and OSTA to predict osteoporosis in Japanese women who underwent hip surgery. In the analysis, the surgeons’ assessment had “moderate” to “good” predictive ability, whereas the OSTA score had “good” predictive ability. When both scores were combined, the AUCs were “good” in 11 surgeons and “excellent” in four surgeons. Collectively, our results indicate that combining surgeons’ assessment and OSTA can be used as a simple convenient screening tool for osteoporosis that can help identify patients who may need further BMD assessment using DXA.

### Comparison of our results with those of previous reports

The effectiveness of using hip X-ray findings in screening for osteoporosis has been reported. For example, the usefulness of parameters, including the Singh index, cortical thickness index, canal flare index, canal-to-calcar ratio, and Dorr classification, has been analyzed. Among them, several studies have reported the usefulness and superiority of the cortical thickness index with an AUC of 0.82–0.84 for diagnosing osteoporosis [12, 27, 28]. In the present study, the AUC from X-ray assessment ranged from 0.626 to 0.875 across surgeons, and the AUC using the ave.pred-score was 0.890, indicating that surgeons’ assessment had high predictive ability to detect osteoporosis from hip X-rays. Conversely, the causality of each surgeon in selecting the pred-score remains unknown and is subjective. However, surgeons likely define their pred-score based on X-ray findings (e.g., cortical thickness and bone structure of the cancellous bone). Although subjective, the intraobserver agreement of the pred-score was “almost perfect,” and no significant difference was found between staff surgeons and residents. Thus, the results support the application of the pred-score in clinical practice regardless of the surgeon’s experience.

With regard to OSTA, previous studies have demonstrated its effectiveness as a screening tool for osteoporosis. Specifically, previous studies have reported that the OSTA has an AUC value ranging between 0.62 and 0.87 [18–22]. In the present study, the AUC of the OSTA score was 0.817, which was included in the range reported previously.

Some studies have reported the usefulness of combining X-ray findings and OSTA. For example, Liu et al. [29] reported that combining the Singh index and OSTA significantly improved the AUC for diagnosing osteoporosis (0.795) compared with using the OSTA (0.534) or Singh index (0.636) alone. While a direct comparison with our study could not be made, the results of our study were likely superior to those of Liu et al’s study, as previous studies have shown the difficulty of using the Singh index in predicting the BMD of the proximal femur.

### Application of the results to clinical practice

In this study, the AUC (0.912) combining the ave.pred-score and OSTA score was significantly improved compared with that of the OSTA score (AUC: 0.817) when analyzed in all surgeons. Alternatively, no significant improvement of AUC was found from the ave.pred-score. When analyzed for each surgeon, the AUC was the highest when the pred-score and OSTA score were combined, with significant improvement from AUCs calculated with the pred-score or OSTA score in 11 surgeons (73.3%). Because X-ray assessment is usually performed by surgeons in their clinical practice (e.g., outpatient clinic), our results support its clinical application regardless of the surgeon’s skill in predicting osteoporosis from hip X-rays. As the method proposed in the present study is not applicable for assessing BMD changes over time, our next step is to clarify factors that enable longitudinal BMD assessment.

There may be other methods to further improve the prediction of osteoporosis screening, including adding other metrics calculated from X-rays (e.g., Singh index, cortical thickness index). However, the addition of several parameters requires time and increases the complexity in measurement and analysis, limiting its use in clinical practice. As “good” or “excellent” AUCs (>0.82) were maintained among all surgeons, we believe that the method developed in this study is clinically useful for osteoporosis screening in patients with hip diseases.

The recent application of artificial intelligence (AI) technology for screening osteoporosis from hip X-rays has considerable attention. In fact, some studies have shown an AUC of >0.9 in detecting osteoporosis from hip X-rays [30–32]. While the “black-box” aspect of AI is a matter of concern, our results may explain why adding patient demographics to the AI model improved the predictive accuracy of osteoporosis [30]. As our study has shown that combining the ave.pre-score and OSTA score maintained an AUC of >0.9, we believe that it would be necessary for future studies to compare the results derived from AI and those derived from other conventional methods in order to clarify the novelty and usefulness of a new AI system.

### Application of the results to patients from other countries and males

Although the OSTA was originally developed for postmenopausal Asian women, studies have confirmed its potential use in osteoporosis screening for men and other ethnicities, including Caucasians and African-Americans [33]. For example, previous studies have reported an AUC of 0.71 for men (94% Caucasians) [34], an AUC of 0.813 for Caucasian women [35], and a sensitivity of 75.4% and specificity of 75.0% with a cut-off value of 2 for African-American women [36]. Thus, while further studies are necessary, we believe that there is a potential for the findings of this study to be tested and validated in different cohorts.

### Limitations

This study has some limitations. First, as information regarding menopause was not available, the patients included in this study were women aged over 40 years. The results may vary if only postmenopausal women were included. Second, the results may not be directly applied to the general population, as the patients analyzed in this study were scheduled for hip surgery due to hip disorders. Although a study acquiring hip X-rays in a healthy population could be conducted, the radiation dose cannot be ignored. As hip X-rays are routinely acquired preoperatively and postoperatively for patients with hip diseases, we believe that the lack of analysis in the general population does not diminish this study’s clinical importance. Third, while hip X-rays were acquired following routine protocol with the lower limb internally rotated, the femur was not always in the neutral position because of its anatomy or disease in the contralateral hip. Our next step may be to clarify the effect of hip rotation on the pred-score of each surgeon because hip rotation may alter the surgeon’s assessment of the femur.

### Conclusions

Surgeon’s X-ray assessment and OSTA had the potential to be used for osteoporosis screening. When the ave.pred-score and OSTA score were combined, the AUC to detect osteoporosis was 0.912. When analyzed for each surgeon, an AUC > 0.82 was maintained across surgeons regardless of their experience when the pred-score and OSTA score were combined. Collectively, our results confirmed that combining the surgeon’s hip X-ray assessment and OSTA is a potentially useful tool to easily screen for osteoporosis and help identify patients who require DXA examination.
